# Children’s drawings as a projective tool to explore and prevent experiences of mistreatment and/or sexual abuse

**DOI:** 10.3389/fpsyg.2023.1002864

**Published:** 2023-02-22

**Authors:** Elisabeth Ballús, Ma Carmen Comelles, Ma Teresa Pasto, Paula Benedico

**Affiliations:** ^1^Department of Psychology, Universitat Ramon Llull, Barcelona, Catalonia, Spain; ^2^Barcelona City Council (Ajuntament de Barcelona), Barcelona, Spain

**Keywords:** children’s drawings, childhood mistreatment, childhood sexual abuse (CSA), the projective Draw-a-Person (D.A.P.), psychometric properties

## Abstract

Reality shows us that situations of mistreatment and sexual abuse in childhood are still seldom visible, despite their high prevalence around the world. It is essential to detect and address them, especially among children in situations of dire risk or neglect. The purpose of this study is to determine if graphic emotional indicators are expressed in the drawings of the projective Draw-a-Person (D.A.P) test, made by children in situations of dire risk or neglect. The sample is made up of 34 children, between the ages of 5 and 11 (17 girls and 17 boys), attended by Specialised Child and Adolescent Care Services of the Barcelona Town Hall (Spain). The drawings were coded quantitatively. The results indicated that most of the drawings show a frequency of graphic emotional indicators, as well as graphic indicators common to experiences of mistreatment and/or abuse, which confirm the existence of emotional problems. However, no significant differences based on gender and age were found, except for one indicator of sexual abuse (body omitted/distorted), which is significantly more common in the boys. Results also revealed that the drawings of human figure enable the children to express their experiences of traumatic situations which are difficult to verbalize. These findings have important implications for professionals, as the use of this projective technique can help to early identification and design treatment strategies in situations of mistreatment and/or abuse in children and their families.

## Introduction

The mistreatment in childhood, understood as physical or emotional abuse, sexual abuse and emotional and physical neglect, is a common experience globally that affects all cultures (Pereda and Abad, 2013; Varese et al., 2013; Stoltenborgh et al., 2014). In the case of childhood sexual abuse (CSA), it is regarded as the biggest public health problem and a violation of human rights (Putnam, 2003; Norman et al., 2012), with serious long-term negative repercussions on individuals’ physical and mental health (Putnam, 2003; Peltzer and Pengpid, 2016; Read et al., 2017). Data published in numerous international studies confirm that abuse in childhood plays a causal role in many adult mental health problems, including depression, anxiety disorders, substance abuse, personality disorders and psychosis (Kendler et al., 2000; Varese et al., 2012; Teicher and Samson, 2013; Read et al., 2017). Paradoxically, most cases of childhood sexual abuse or neglect are not identified by the mental health services (Read et al., 2017).

There is also evidence that experiencing multiple kinds of abuse over a period of time increases the risk of developing psychological and emotional problems compared to children who have only experienced occasional abuse (Warmingham et al., 2019). Indeed, childhood is an essential, highly sensitive period in human development. Marques-Feixa and Fañanás (2020) recently showed that mistreatment of children plays a crucial role in [arresting] individuals’ neurobiological and psychic maturation. The same authors also state that when this abuse arises in childhood, it deregulates several neurobiological and stress-regulatory systems that are essential in the consolidation of complex cognitive and emotional-regulating functions. These systemic changes can make individuals more vulnerable to suffering from different mental disorders and other medical conditions during childhood and adulthood.

The data from global studies on child sexual abuse (CSA) and its prevalence around the world seem to concur that it occurs persistently (Pereda et al., 2009; Stoltenborgh et al., 2011; Nguyen et al., 2017). According to the World Health Organisation (WHO, 2016), around the world 20% of women and 5–10% of men claim to have suffered from sexual abuse as children. Likewise, the differences in geographic regions of the world with different beliefs and cultural values may affect the estimated incidence of CSA (Kenny and McEachern, 2000). For instance, studies conducted in Asian countries show a lower prevalence of CSA than in non-Asian countries, which could be attributed to conservative cultural sexual norms (Elliott and i Urquiza, 2006; Nguyen et al., 2010). Furthermore, different meta-analyses (Pereda et al., 2009; Stoltenborgh et al., 2011) report a higher prevalence of CSA among girls than boys. However, other times the sex of victims of abuse depends on the specific context where the abuse occurs; for example, in the case of victims of clergy abuse, the vast majority of victims are males (Faller, 2020). In recent decades has been increasing public attention to the child abuse (CSA) occurring within civic institutions, such as school setting, youth sports, religious institutions and other youth service child and youth-serving organizations (Harris and Terry, 2019).

Despite the greater awareness and social concern for the mistreatment, neglect and sexual abuse among children today, one of the major difficulties in bringing visibility to it is detection (Tello, 2020). As some research indicates, the majority of children do not disclose their sexual abuse during childhood (London et al., 2005, 2008). Keeping their sexual abuse to themselves, often leading to more several mental health and other detrimental consequences than if they had disclosed (Faller, 2020). This information revels the importance of having instruments for children that could to help them to express their traumatic experiences. The majority of instruments used to evaluate sexual abuse in children are interviews and self-reported questionnaires, like “The Adverse Childhood Experiences International Questionnaire” (ACE-IQ). The ACE-IQ is designed to be administered to adults aged 18 years and older and to assess childhood adversities worldwide. It is comprised three categories of child abuse (psychological, physical and sexual contact), two categories of neglect and four categories indicative of exposure to household dysfunction (Pace et al., 2022b). It primarily uses interviews to assess children, despite the limitations in verbal expression at that age and the traumatic situation itself. Drawing has usually been used in clinical contexts, and the use of drawing with children who have experienced sexual abuse is considered extremely important because it helps them express their emotions more freely (Cohen-Liebman, 1999; Kissos et al., 2019). There is a need to create more sensitive interviews so that children will not relive the trauma or experience new trauma *via* intrusive or inappropriate interviews (Katz et al., 2014; Lev-Wiesel et al., 2021). Therefore, drawings can be included in interviews, but they are assessed more qualitatively and often lack a scoring system. In short, there is a lack of instruments to assess CSA in childhood.

### The current study

In order to contextualize the present study, we will explain briefly how the Child Protection System in Catalonia (Spain) is organized. One out of every five people in Catalonia (Spain) has suffered some type of sexual violence during their childhood, according to the “Save the Children” report entitled *Ulls que no volen veure* [Eyes that do not want to see] (2017), Currently, the Barcelona Town Hall manages the child and adolescent care service comprised of specialised interdisciplinary teams, SEAIA (Specialised Child and Adolescent Care Services), made up of professionals from the fields of psychology, pedagogy, social work and social education. Its purpose is to attend to children in situations of dire risk and/or neglect. Situations of serious risk mean circumstances in which the development and wellbeing of children and adolescents are limited or harmed by some personal, social or family situation, provided that the effective protection of such children or adolescents does not require separation from the nuclear family (LDOIA, Law 14/2010, on Rights and Opportunities for Children and Adolescents in Catalonia). On the other hand, the same law defines situations of neglect as those where children or adolescents find themselves in a situation where they lack the basic necessities for the comprehensive development of their personality, provided that their protection does require the application of a measure involving separation from the nuclear family. SEAIA’s mission is divided into four areas of intervention: (1) Individualised assistance for children/adolescents and their families (diagnostic study, monitoring the development of the child and their family through support and other interventions), (2) Advice/Collaboration with Basic Social Services, (3) Community work, and (4) Institutional collaboration. Regarding the instruments currently used in these teams are interviews and questionnaires geared at families and caregivers, such as the Adult Attachment Interview (Barudy and Dantagnan, 2010) and the Questionnaire to Evaluate Adopters, Caregivers, Guardians and Mediators (CIUDA, Bermejo et al., 2014). For children, interviews are held and drawings are used, although they are not coded. Therefore, it is essential to have objective, valid, reliable tools that are age-appropriate in order to detect and/or confirm situations of mistreatment and/or sexual abuse, which are so difficult to express verbally.

### Drawings and projective methods

There is a consensus in regarding drawings as a non-intrusive technique (Jacobs-Kayam et al., 2013; Kissos et al., 2020), given that drawing is a natural and spontaneous language for children, as it is ontologically and genetically more primitive than writing and does not require special training (Ballús and Viel, 2007). This study has used drawings of human figures by children as a graphic projective tool within the conceptual framework of psychoanalysis to facilitate non-verbal expression of the children’s traumatic experiences. According to Piaget and Inhelder (1920), drawing involves the externalisation of a previously internalised mental image, which projects the individual’s internal worlds onto external spaces (Siquier de Ocampo et al., 1987). Therefore, it provides access to the unconscious (Frank, 1939). According to this same author, projective techniques provide an approach to an individual’s personality and persuade them to reveal how their experience is organised, giving them few guidelines or little structure (the instructions in this study: “Draw a person”), so that their personality and especially their feelings can be projected (Avila, 1997). The subject is considered to project their self-concept, which is constructed from each individual’s subjective experiences (Schilder, 1935). It is not developmental but instead is unique to and characteristic of each person. Projective techniques, however, are understood to be partial tools in a comprehensive diagnostic process.

While it is true that graphic projective techniques have often been used in the clinical area, in research (such as Machover, 1953; Koppitz, 1968; Hammer, 1978; Bellak, 1979; Frank de Verthelyi and Rodríguez, 1985; Wohl and Kaufman, 1985; Buck, 1995) and in the field of sexual abuse (Wohl and Kaufman, 1985; Van Hutton, 1994; Royer, 1999; Colombo et al., 2004; Maganto and Garaigordobil, 2009b; Pont, 2012), the psychometric requirements of reliability and validity have been one of these tools’ most controversial issues (Avila, 1997; Maganto and Garaigordobil, 2011). Projective techniques, and analyses of drawings in particular, initially put the emphasis solely on qualitative analysis, thereby undermining the data’s objectiveness and validity. The lack of methodological rigour in the use of drawings in psycho-diagnoses in the 1960s and 1970s resulted in the techniques being criticised and disparaged (Maganto and Garaigordobil, 2011; Allen and Tussey, 2012). However, according to Ballús et al. (2020), over the last few years there has been a proliferation of research using projective methods while incorporating quantitative measures with psychometric properties (Maganto and Garaigordobil, 2009b; Barbosa and Sales, 2014, 2015; Tuset and Fernández, 2017; Ballús et al., 2019a,b), adding objectivity and reliability to these methods.

On the other hand, although it is true that the literature review concludes that there is no evidence that drawings can be used as a valid indication of personality or for diagnosis, some scoring systems may be adequate for screening purposes (Goldner et al., 2018). Different scoring systems have been developed based on the Draw-A-Person (DAP) test by Machover (1953), such as the DAP-SPED scoring system (Naglieri et al., 1991). It uses an objective approach based on the frequency of items depicted in human figure drawings that are considered indicators of possible emotional problems in non-clinical versus clinical populations. Similarly, Maganto and Garaigordobil (2009b) developed and validated a psychometric DAP test, the Two Human Figures test (T2F), which gives the test validity and reliability with its scoring system to identify developmental and emotional indicators (some of them common to experiences of sexual abuse) in children aged 5 to 12. The test is both quantitative and qualitative, enables the emotional indicators to be coded and makes it possible to determine whether or not emotional problems are present according to the percentile obtained, while it also offers a more qualitative analysis of the meaning of the emotional indicators found. Moreover, in the research on drawings often is questioned the discriminant validity with the results obtained (in this case with the T2F) and the drawing ability of the child (Clarke et al., 2002; Pace et al., 2022c). Regarding the Two Human Figures test (T2F), the items of Developmental Indicators scale (52 items) was developmental (Maganto and Garaigordobil, 2009b). That is, its frequency in drawing increases as the subjects get older. It is based on assigning standardised scores when development indicators are present, and the resulting value is then transformed into percentiles for each age and sex. That’s means, the T2F test classifies and situates the child’s drawing in reference to his or her normative group, based on age and gender. In addition, these items correlate with intelligence evaluated by Raven (1995).

In terms of the empirical evidence to detect sexual abuse in self-figure drawings, as Kissos et al. (2020) state, in the past 20 years different studies have shown that the drawings in DAP tests by individuals who are the victims of sexual abuse have specific graphic features that differ from those drawn by persons who have not been abused (Faller, 2014). Previous studies have suggested that the omission or distortion of body parts in self-figure drawings implies conflictive relationship with the part and are associated with trauma and abuse (Koppitz, 1968; Dyer et al., 2015). For instance, in trauma and abused victims’ self-figure drawings, the whole body or certain body parts are omitted or distorted (Jacobs-Kayam et al., 2013; Dyer et al., 2015; Goldner and Frid, 2021), and there are other indicators as well, such as the head detached or disconnected from the body (Faller, 2003; Handler and Thomas, 2013; Goldner and Frid, 2021). Moreover, other studies also provide validation of four indicators of sexual abuse (Jacobs-Kayam et al., 2013) that have previously been documented (Lev-Wiesel, 1999; Amir and Lev-Wiesel, 2007): (1) the face line, (2) the eyes, (3) the hands and arms, and (4) the genitals. The presence of three or more of these features is considered to indicate sexual abuse. Furthermore, recent studies (Goldner et al., 2021) code the drawing style with some of the following indicators of sexual abuse: pre-schematic drawing; size of figure: small (about 2 cm) or oversized; and presence of aggressive symbols.

The present study, in line with the latest research projects, uses graphic projective techniques incorporating not just the qualitative analysis characteristic of these techniques but also quantitative analysis through the codification of several graphic indicators. The main aim of this research is to determine whether graphic emotional indicators, including those of child sexual abuse (CSA), were expressed in the drawings of the projective DAP test made by children in situations of dire risk or neglect. We have formulated two hypotheses based on previous findings: First (H1), graphic indicators of child sexual abuse (CSA) will be found in the drawings of the children in the sample, in situations of dire risk or neglect (Jacobs-Kayam et al., 2013; Dyer et al., 2015; Goldner et al., 2021). Second (H2), more than a half of the participants will have a highest frequency of emotional indicators, corresponding to the upper percentiles on the emotional scale of the Two Human Figures test (T2F), confirming the existence of emotional problems (Magannto and Garaigordobil, 2009b).

## Methods

### Participants and procedure

The sample is made up of 34 children in situations of serious risk or neglect who in 2018 were receiving care from Barcelona’s Specialised Childcare Services (SEAIA) in Catalonia (an autonomous community in northeast Spain, which has 16% of the total national population).

The 34 children in this study range in age from 5 to 11. The sample consisted of 17 girls and 17 boys with a mean age of 7.91 (SD = 1.6). Two age groups were created to facilitate data analysis in accordance with the authors of one of the instruments used, the Two Human Figures test (T2F) by Maganto and Garaigordobil (2009b). These authors stated that the majority of the emotional indicators are common to all ages, but some are only significant after the age of 7. Therefore, two age groups were made, matching this division. The first group was aged 5–7 (*n* = 14) and consisted of 8 girls and 6 boys, with a mean age of 6.36, and the second group was aged 8–11 (*n* = 20) and made up of 9 girls and 11 boys, with a mean age of 9 years.

The children in the sample were chosen at random from the child population receiving care from by Barcelona’s SEAIA in 2018. According to the data provided by the Catalan government’s Directorate General of Child and Adolescent Care (2018), the number of children and adolescents in the population was 1,402,825. In December of that year, 18,262 children and adolescents were receiving assistance under the protection system. Of them, 8.672 (47.4%) were involved in an intervention with the family without separate, while the remaining 9,590 (52.6%) had a protection measure in place involving separation from their nuclear family. Specifically, 3,742 children (39%) were subject to a family-foster care measure (65.2% with extended family, 24.2% in foster families and 10.4% in pre-adoption foster care); other 5,681 children (59.2%), were in residential care, and the 167 remaining children (1.7%) were in other situations (hospital, juvenile justice, etc.).

The project was approved by the Ethics and Research Committee at the Universitat Ramon Llull (URL) in Barcelona (Spain). In accordance with the professional conduct regulations, signed parental consent and personal data protection were obtained. Likewise, to ensure the anonymity of the personal information of the children in the research, the subjects were assigned an identification number and only their age and sex were stated. The data were collected at SEAIA’s Barcelona office. The test was administered to the children individually by the SEAIA staff, who had previously been trained by the principal investigator. Moreover, their attitudes and reactions to the test were noted. Afterwards, two members of the research team specialising in projective techniques analysed the test.

### Measures

#### Indicators of childhood sexual abuse in human figure drawings

Based on the empirical evidence from recent studies on the specific graphic characteristics presented by the human figure drawings (DAP) of sexually abused children (Lev-Wiesel, 1999; Amir and Lev-Wiesel, 2007; Jacobs-Kayam et al., 2013; Dyer et al., 2015; Kissos et al., 2019; Goldner and Frid, 2021; Goldner et al., 2021), new indicators have been taken into account for this study.

The indicators of child sexual abuse (CSA) used to assess the human figure drawings are as follows: (1) whole body or body parts are omitted or distorted (Jacobs-Kayam et al., 2013; Dyer et al., 2015; Goldner and Frid, 2021), (2) the head is detached or disconnected from the body (Faller, 2003; Handler and Thomas, 2013; Goldner and Frid, 2021), (3) the face line is double or hollow, or the chin or cheek are shaded (Lev-Wiesel, 1999; Amir and Lev-Wiesel, 2007; Jacobs-Kayam et al., 2013), (4) the eyes are in the form of dots, hollowed, shaded or omitted (Lev-Wiesel, 1999; Amir and Lev-Wiesel, 2007; Jacobs-Kayam et al., 2013), (5) the hands and arms are depicted as clinging, detached, cut off or are omitted (Lev-Wiesel, 1999; Amir and Lev-Wiesel, 2007; Jacobs-Kayam et al., 2013), (6) the genitals are shaded or blocked off from the rest of the body (Lev-Wiesel, 1999; Amir and Lev-Wiesel, 2007; Jacobs-Kayam et al., 2013), (7) pre-schematic drawing (blocked human figures, primitive figures corresponding to ages 4–5; Goldner et al., 2021), (8) the size of the figure: small (about 2 cm) or oversized such that the figure occupies most of the page (Goldner et al., 2021), and (9) the presence of aggressive symbols (Goldner et al., 2021).

#### The projective two human figures test

The instrument used was the Two Human Figures test (T2F) of Maganto and Garaigordobil (2009b). It’s a psychometric proposal for the graphic projective test Draw-A-Person (DAP), from the developmental and projective perspectives giving it greater validity and reliability. The scoring system is based on frequency of items in a human drawing. Which is described for the authors, as a screening instrument to be used in clinical, educational and social settings to identify children with developmental (52 Indicators) and emotional problems (35 Indicators). For the purpose of this study, the drawings were coded only using the 35 Emotional Indicators scale. Participants were asked to draw a person, on a sheet of Din A4 paper which they had been handed previously, along with a pencil and rubber and with no time limit. Once they had finished, they were given a second sheet of paper and is requested to draw a person of the opposite sex. For the youngest children, the instruction was to draw a boy or girl, according with Machover (1953).

Using Spanish samples of 1,222 and 1,623 participants aged 5 to 12, results showed that the instrument was both reliable and valid for to identify developmental and emotional problems (Maganto and Garaigordobil, 2009a). Regarding the Developmental Indicators scale (52 items), it is based on assigning standardised scores when development indicators are present, and the resulting value is then transformed into percentiles for each age and sex. Two criteria to accept these items were agreed upon: (1) the item was developmental; that is, its frequency in drawing increases as the subjects get older and (2) it correlates with intelligence evaluated by Raven (1995). To check this, contingency analyses were performed by calculating the Chi-square by ages and age groups for both the male and the female human figure drawings, and Pearson correlations were performed between the scores earned on the T2F and Raven. The results revealed significant correlations (*p* < 0.05) between the variables, confirming the validity of the test. The Cronbach’s coefficient (0.86) and the Spearman-Brown (0.86) were also calculated and found satisfactory.

Regarding the Emotional Indicators scale (35 items), these Emotional Indicators meet three criteria (Tuset and Fernández, 2017): (1) they distinguish between clinical and non-clinical groups, (2) they are not developmental, and (3) they are unusual at any age (frequency under 10%). Sixty indicators were initially chosen, but the Chi-squared contingency analysis of Pearson for each of the figures, between the clinical and non-clinical sample, concluded that statistically significant differences were only found in 35 of the emotional indicators. Furthermore, the analyses performed between emotional items and age showed a negative covariation, in that as development advances, the representation of those emotional indicators drops. This enabled us to conclude after what age these items should be considered emotional indicators. Therefore, of the 35 emotional indicators, 23 indicators are common to all ages, 6 indicators are applied from the age of 7 onwards and another 6 indicators from the age of 9 onwards.

These Emotional Indicators, according to the T2F’s authors, need to be interpreted with two complementary aspects taken into account: (1) Number of indicators and (2) Types of emotional indicators present. As for (1) Number of indicators, the assessment is quantitative based on the application of cut-off points according to the percentile, aforementioned (Table 1), which determine whether or not the subject presents emotional problems (*75th percentile:* points to the possible existence of emotional problems; *85th percentile:* considered a high level of probability of the existence of emotional problems*; 95th percentile:* confirms the existence of emotional problems). The assessment of (2) types of indicators, is qualitative based on the review of literature from experts in the field carried out by Maganto and Garaigordobil (2009b). Moreover, the authors also point out that some emotional indicators have particular clinical-emotional relevance. Within the 23 indicators common to all ages, these indicators are the following: (1) Bizarre, unreal, grotesque or monster figure, (14) Genitals or sexual characteristics, (19) No eyes, (20) No mouth, and (21) No body. And the six indicators applied from the age of 7, include the following: (25) Leaning figure.

**Table 1:** Conversion of directs scores form the T2F-E test to percentiles according to age (Maganto and Garaigordobil, 2009b).

Percentiles	5 years old	6 years old	7 years old	8 years old	9 years old	10 years old	11 years old	12 years old
99	6 or >	7 or >	8 or >	9 or >	9 or >	10 or >	10 or >	11 or >
95	4–5	4–6	6–7	6–8	6–8	7–9	7–9	8–10
85	3	3	5	5	5	5–6	6	6–7
75	2	2	4	4	4	4	5	5
<75	0–1	0–1	0–3	0–3	0–3	0–3	0–4	0–4

95th and 99th percentile: Existence of emotional problems; 85th percentile: High probability of emotional problems; 75th percentile: Possible existence of emotional problems; < 75th percentile: No emotional problems.

## Data analysis

The data were analysed using the JASP statistical programme (version 0.16.3). First, to analyse indicators of sexual abuse (CSA), the descriptive statistics (mean and standard deviation) of the sample and the frequencies and percentages of the indicators were calculated. Then, the chi-square (χ2) was conducted to carry out a comparative study with the results obtained (presence or absence of indicators) based on gender. Finally, the descriptive statistics (mean and standard deviation) of the sample and the frequencies of the emotional indicators in Two Human Figure test (T2F), were used.

## Results

The results that presented below are intended to respond to the hypotheses raised. For the first hypothesis (H1), on whether graphic indicators characteristic of child sexual abuse (CSA) will be present in the drawings of the children in the sample. Only the first human figure drawings were used. As for the second hypothesis (H2), more than a half of the participants will have a highest frequency of emotional indicators, corresponding to the upper percentiles on the emotional scale of the Two Human Figures test (T2F), confirming the existence of emotional problems. In this case, both humans figure drawings (first and latter of the opposite sex), were taken into account.

### Indicators of sexual abuse in human figure drawings

**Figure 1:**
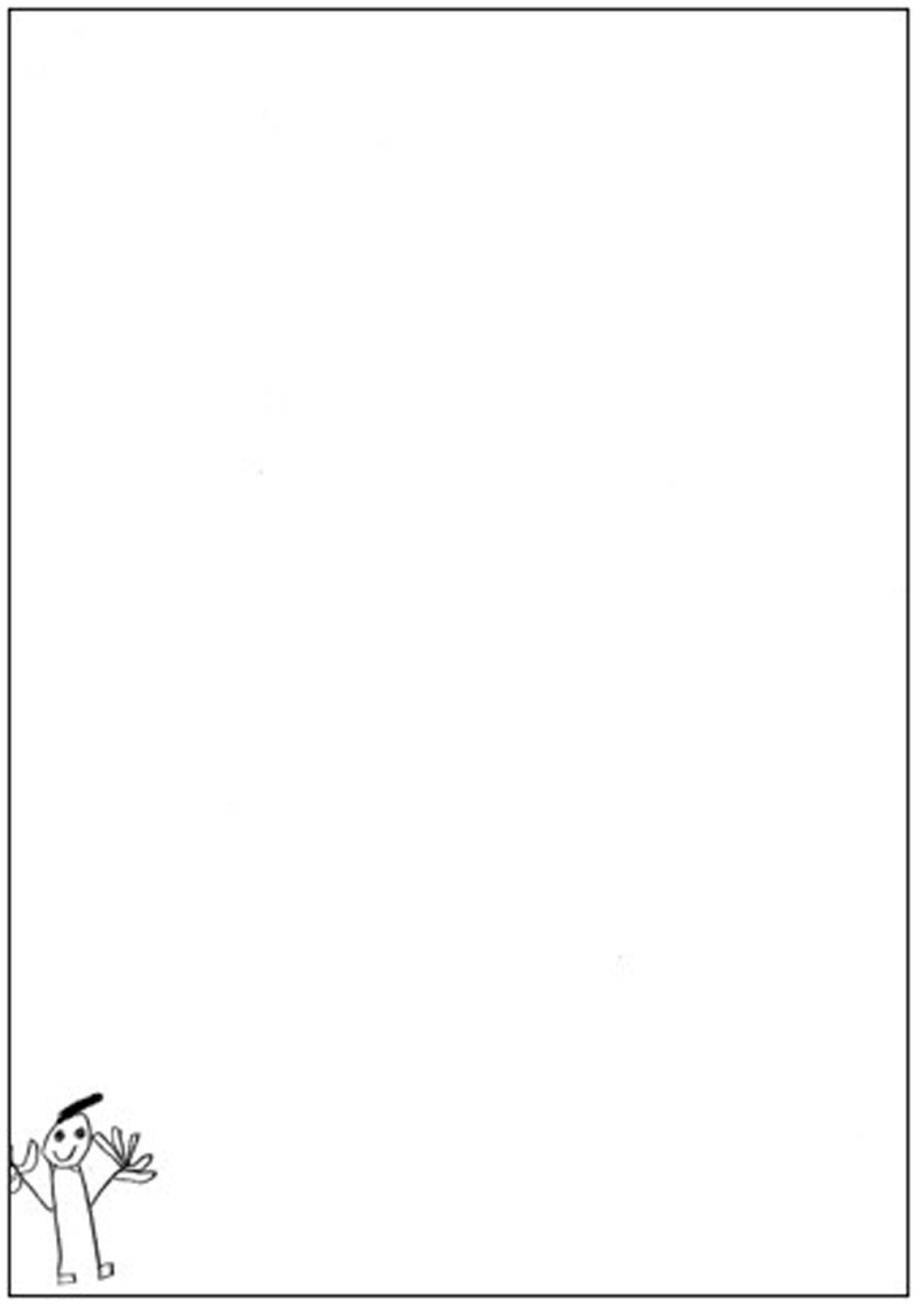
Subject 16: First drawing (male figure) of 7-year-old boy. CSA indicators: Indicator 1 (omission of body), Indicator 4 (dot/shaded eyes), Indicator 7 (pre-schematic drawing) and Indicator 8 (small figure size).

Chi-Squared analyses were conducted to identify associations with indicators of sexual abuse and the gender of the participants. The results of our study revealed gender differences between the participants. As shown in Table 2, there is a significant difference between boys and girls (χ2 = 4.250; *p* = 0.039*) in Indicator 1. Body omitted/distorted (Omission or distortion of the entire body or parts of the body). The boys showing a greater presence of the item, that’s mean, 70.6% the boys versus 35.3% the girls. It should be noted that this Indicator, 1 Body omitted/distorted, has been the indicator of sexual abuse CSA with the most frequency (52.9%; *n* = 18), This occurred in over half of the subjects based on the distortion (*n* = 8) or omission of the entire body (*n* = 5) or parts of the body (*n* = 5). Otherwise, no significant differences were found between gender or age in the other indicators. Illustration 1 (see Figure 1), was the first drawing (male figure) by a 7-year-old boy. This is an example of Indicator, 1 Body omitted and other CSA indicators as Indicator 4 (dot/shaded eyes), Indicator 7 (pre-schematic drawing) and Indicator 8 (small figure size).

**Table 2:** Frequencies of indicators of sexual abuse in human figure drawings (CSA).

Indicators of CSA		Children aged 5–11 (*n* = 34)						
		Female (*n* = 17)		Male (*n* = 17)		Total Present	Chi-Square	Value of *p*
		Present	Absent	Present	Absent			
1. Body omitted / distorted	*n*	6	11	12	5	18	4.250	0.039*
	%	35.3	64.7	70.6	29.4	52.9		
2. Head	*n*	2	15	2	15	4	0.000	1.000
	%	11.8	88.2	11.8	88.2	11.8		
*3. Face line*	*n*	1	16	3	14	4	0.283	0.595
	%	5.9	94.1	17.6	82.4	11.8		
*4. Eyes*	*n*	7	10	7	10	14	0.000	1.000
	%	41.2	58.8	41.2	58.8	41.2		
*5. Hands/arms*	*n*	8	9	4	13	12	2.061	0.151
	%	47.1	52.9	23.5	76.5	35.3		
*6. Genitals*	*n*	1	16	0	17	1	0.000	1.000
	%	5.9	94.1	0	100	3		
7. Preschematic drawing	*n*	3	14	4	13	7	0.000	1.000
	%	17.6	82.4	23.5	76.5	20.6		
8. Size of figure	*n*	3	14	2	15	5	0.000	1.000
	%	17.6	82.4	11.8	88.2	14.7		
9. Aggressive symbols	*n*	3	14	3	14	6	0.000	1.000
	%	17.6	82.4	17.6	82.4	17.6		

*p < 0.05. The presence of three or more indicators in italic (Indicator 3, 4, 5, 6), is considered to indicate sexual abuse (Jacobs-Kayam et al., 2013).

Descriptive statistics (mean and standard deviation) of the number of indicators presented in each of the individuals were calculated according to whether they were boys or girls. The results of frequency of indicators of CSA show that there are no differences between boys (*M* = 2.18; SD = 1.24) and girls (*M* = 2; SD = 1.41). Moreover, the results showed than more than half of the drawings (61.7%;) present between two and five indicators. Specifically, 44.1% show between two and three indicators and 17.6% show between four and five indicators. However, in no case were indicators 3, 4, 5 and 6, which indicate sexual abuse, obtained at the same time (Jacobs-Kayam et al., 2013).

In addition, as shown in Table 2, the most frequent Indicators of Sexual Abuse (CSA) are three: Indicator 1. Body omitted/distorted (52.9%; *n* = 18), Indicator 4. Eyes (41.2%; *n* = 18). They represented eyes with a single dot (*n* = 6), with shading (*n* = 4) or hollow (*n* = 4). Likewise, Indicator 5. Hands/arms (35.3%; *n* = 12), were represented as detached (*n* = 2) or omitted (*n* = 10).

Nevertheless, 20.6% of the sample produced a pre-schematic drawing (Indicator 7); that is, they drew a primitive figure corresponding to one that a child aged 3 or 4 would make. As for the presence of aggressive symbols (Indicator 9), they were observed in 17.6% of the sample, more specifically with the expression of teeth (*n* = 4), nails (*n* = 1) and weapons and blood (*n* = 1). As for the size of the figures (Indicator 8), 14.7% of the participants drew either very small figures, smaller than 2 cm (*n* = 1) or very large figures (*n* = 4). Indicator 2 (Head detached from rest of body) was observed in 11.8% of the drawings, the same as Indicator 3, (double face outline). Finally, we should note that only 3% of our sample drew genitals (Indicator 6).

### Emotional indicators in projective two-human-figure drawings

First of all, we should point out that 94.12% of this study’s subjects drew a human figure of their own sex first, whereas 5.88% drew a figure of the opposite sex.

Based on the Two-Human-Figure drawings (T2F), the results were interpreted from two standpoints according to the T2F’s authors: (1) a quantitative analysis on the number of indicators based on the application of the cut-off points mentioned above, and (2) a qualitative analysis referring to the type of emotional indicators found in the sample based on the review of the expert literature on the topic conducted by Maganto and Garaigordobil (2009a).

#### Quantitative analysis (number of emotional indicators)

To perform the quantitative analysis of the results, the frequency of indicators of each individual in each of his or her drawings, both the female and male figure, was calculated along with their corresponding percentages, following the instructions proposed by Maganto and Garaigordobil (2009a) in Table 1. Moreover, the descriptive statistics (mean and standard deviation) each of the percentiles was calculated. Regarding the frequency and type of emotional indicator, no differences were found by sex or age. As regards the number of emotional indicators in the sample, the presence of emotional problems is confirmed in 52.94% of the participants in this study (Table 3), as they received scores equal to or above the 95th and 99th percentiles. Each of these subjects presented an average of 5.89 and 9.33 indicators between the male and female figures drawings. Nevertheless, 20.59% showed a high level of probability of presenting emotional problems, given that they obtained scores equal to or above the 85th percentile. In this case, each of these subjects presented an average of 4.71 indicators between the male and female figure drawings. In addition, 8.82% of the subjects may present emotional problems, as they obtained scores equal to or above the 75th percentile. Four emotional indicators were recorded in each of the three subjects in the male and female figure drawings. By contrast, 17.65% of the sample presented no emotional problems, as they obtained scores below the 75th percentile, with an average of 1.17 indicators between the male and female figures.

**Table 3:** Total frequency of Emotional Indicators in Two Human Figure test (T2F).

Percentiles	Female (*n* = 17)		Male (*n* = 17)		Total (*n* = 34)			
	*n*	%	*n*	%	*n*	%	*M*	SD
99	4	23.53	5	29.41	9	26.47	9.33	1.73
95	6	35.29	3	17.65	9	26.47	5.89	1.05
85	2	11.76	5	29.41	7	20.59	4.71	1.25
75	2	11.76	1	5.88	3	8.82	4	0
< 75	3	17.65	3	17.65	6	17.65	1.17	0.98

95th and 99th Percentiles: Existence of emotional problems; 85th Percentile: High probability of emotional problems; 75th Percentile: Possible existence of emotional problems; < 75th Percentile: No emotional problems.

#### Qualitative analysis (types of emotional indicators)

To perform the qualitative analysis, the frequency and percentage of the different types of emotional indicators were calculated, which Maganto and Garaigordobil (2009b) divide into three age groups: (1) Emotional indicators common to all ages (5–11), (2) Emotional Indicators from age 7, and (3) Emotional Indicators from age 9.

**Figure 2:**
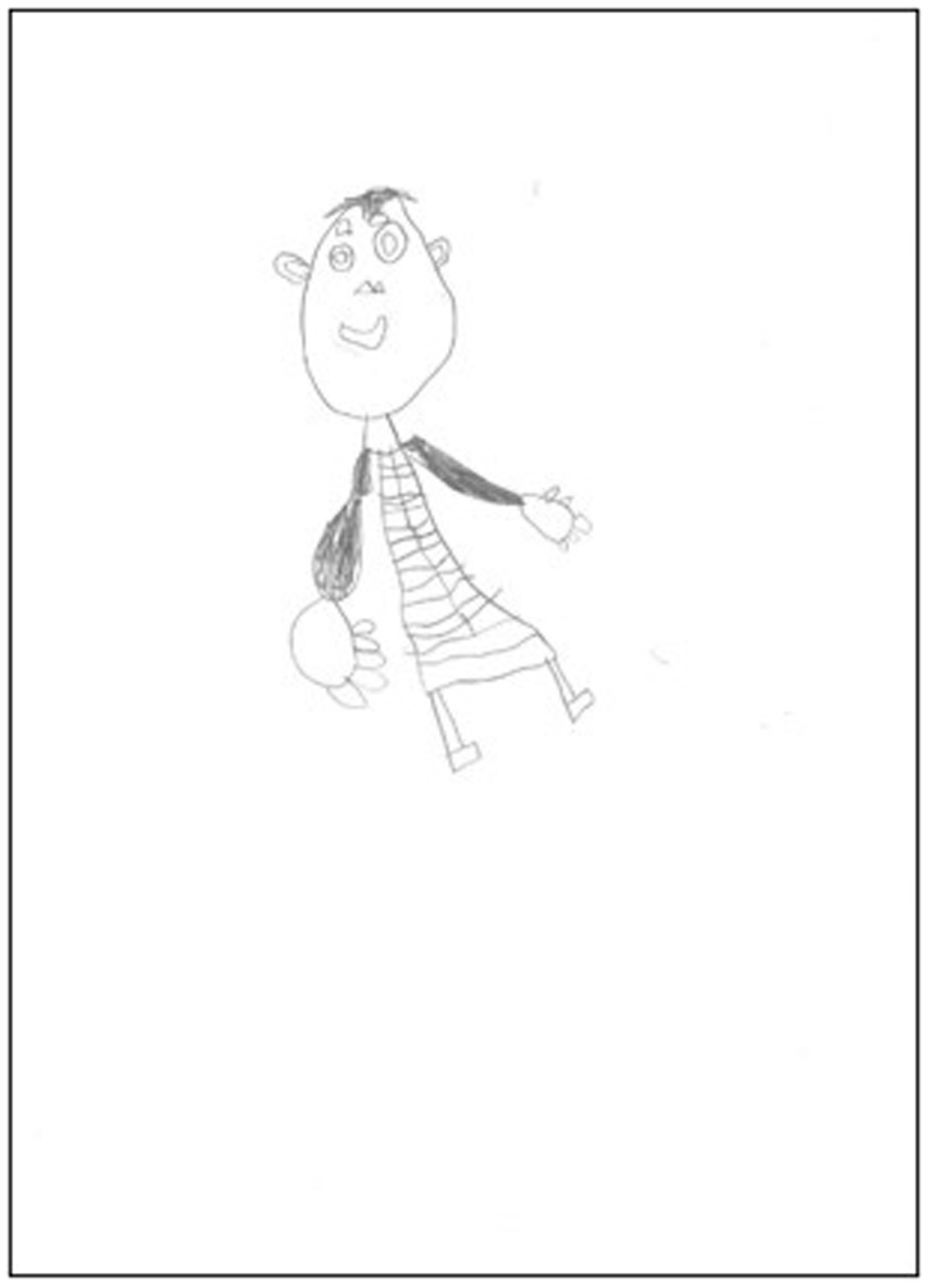
Subject 31: First drawing (female figure) of 7-year-old girl, suspected of having been abused. T2F emotional indicators: Indicator 2 (asymmetric limbs), Indicator 12 (big hands or fingers), Indicator 18 (limbs shaded) and Indicator 25 (leaning figure).

First, Table 4 shows the results obtained common to first group (1) Emotional indicators common to all ages in the sample (*n* = 34). The most frequent indicators were Indicator 2. asymmetric limbs, at 20.6%; Indicator 12. big hands or fingers, which is suggestive of sexual abuse, at 19.1% (Figure 2); and Indicator 21*, omission of body, which has special clinical relevance, at 14.7%. However, note Indicator 1*, bizarre, unreal, grotesque or monster figure, which has special clinical relevance, is present in 10.3%. Furthermore, other indicators suggestive of sexual abuse were found, such as Indicator 14, genitals or emphasised sexual features, which was found in 7.4% of the sample; Indicator 7, transparencies in 5.9% and Indicator 18, limbs shaded in 5.9% of the sample. Illustration 2 (see Figure 2), was the first drawing (female figure) by a 7-year-old girl, suspected of having been abused. This is an example of the next T2F emotional indicators: Indicator 2 (asymmetric limbs), Indicator 12 (big hands or fingers), Indicator 18 (limbs shaded) and Indicator 25 (leaning figure).

**Table 4:** Frequencies of emotional indicators common to all ages in Two Human Figure test (T2F).

Emotional indicators	Children aged 5–11					
	Male drawing (*n* = 34)		Female drawing (*n* = 34)		Total (*n* = 68)	
	*n*	%	*n*	%	*n*	%
1. Bizarre, unreal, grotesque or monster figure (*)	4	11.8	3	8.8	7	10.3
2. Asymmetric limbs	8	23.5	6	17.6	14	20.6
3. Cut figure	2	5.9	2	5.9	4	5.9
4. 2 or more figures	1	2.9	3	8.8	4	5.9
5. Enclosed or framed figure	0	0	2	5.9	2	2.9
6. Big figure	3	8.8	4	11.8	7	10.3
*7. Transparencies*	2	5.9	2	5.9	4	5.9
8. Crossed or wandering eyes	4	11.8	1	2.9	5	7.4
9. Teeth	3	8.8	4	11.8	7	10.3
10. Long arms	4	11.8	3	8.8	7	10.3
11. Arm extensions	2	5.9	1	2.9	3	4.4
*12. Big hands / fingers*	6	17.6	7	20.6	13	19.1
13. Nails	1	2.9	2	5.9	3	4.4
14. Genitals or sexual characteristics (*)	1	2.9	4	11.8	5	7.4
15. Big feet	3	8.8	2	5.9	5	7.4
16. Face shading	5	14.7	1	2.9	6	8.8
*17. Body shading*	2	5.9	2	5.9	4	5.9
*18. Limb shading*	2	5.9	2	5.9	4	5.9
19. No eyes (*)	0	0	0	0	0	0
20. No mouth (*)	0	0	0	0	0	0
21. No body (*)	5	14.7	5	14.7	10	14.7
22. No arms	3	8.8	3	8.8	6	8.8
23. No legs	1	2.9	4	11.8	5	7.4

Frequencies of emotional indicators common to all ages in Two Human Figure test (T2F).

(*) Special clinical relevance indicators (Maganto and Garaigordobil, 2009b).

Indicators in italic: Suggestive of sexual abuse (Royer, 1999; Pont, 2012).

Second, Table 5 shows the results to second group (2) Emotional Indicators from age 7, (*n* = 28). First, the most frequent item among these subjects was Indicator 28, addition of 3 or more details, at 16.1%. Moreover, Indicator 26, hands cut off, which is suggestive of sexual abuse, was observed in 12.5% of the subjects.

**Table 5:** Frequencies of emotional indicators from age 7 in Two Human Figure test (T2F).

Emotional indicators	Children aged 7–11					
	Male drawing (*n* = 28)		Female drawing (*n* = 28)		Total (*n* = 56)	
	*n*	%	*n*	%	*n*	%
24. Poorly integrated figure	3	10.7	4	14.3	7	12.5
25. Leaning figure*	1	3.6	2	7.1	3	5.4
*26. Hands cut off*	2	7.1	5	17.9	7	12.5
27. No feet	3	10.7	4	14.3	7	12.5
28. 3 or more details	5	17.9	4	14.3	9	16.1
29. Intense erasing or second attempt	4	14.3	4	14.3	8	14.3

(*) Special clinical relevance indicators (Maganto and Garaigordobil, 2009b).

Indicators in italic: Suggestive of sexual abuse (Royer, 1999; Pont, 2012).

Finally, the results shown in Table 6 demonstrate the emotional indicators to third group (3) Emotional Indicators from age 9 (*n* = 12). Indicator 34, omission of nose, was recorded in 29.2% of the drawings by the children aged between 9 and 11. Nevertheless, indicators suggestive of sexual abuse, such as Indicator 30, tiny figure, were found in 12.5% of the participants, and Indicator 32, empty eyes, was found in 16.7% of the subjects.

**Table 6:** Frequencies of emotional indicators from age 9 in Two Human Figure test (T2F).

Emotional indicators	Children aged 9–11					
	Male drawing (*n* = 12)		Female drawing (*n* = 12)		Total (*n* = 24)	
	*n*	%	*n*	%	*n*	%
*30. Tiny figure*	*1*	*8.3*	*2*	*16.7*	*3*	*12.5*
31. Big head	0	0	0	0	0	0
*32. Empty eyes*	2	16.7	2	16.7	4	16.7
33. Short arms	2	16.7	2	16.7	4	16.7
34. No nose	3	25	4	33.3	7	29.2
35. No neck	3	25	3	25	6	25

Indicators in italic: Suggestive of sexual abuse (Royer, 1999; Pont, 2012).

**Figure 3:**
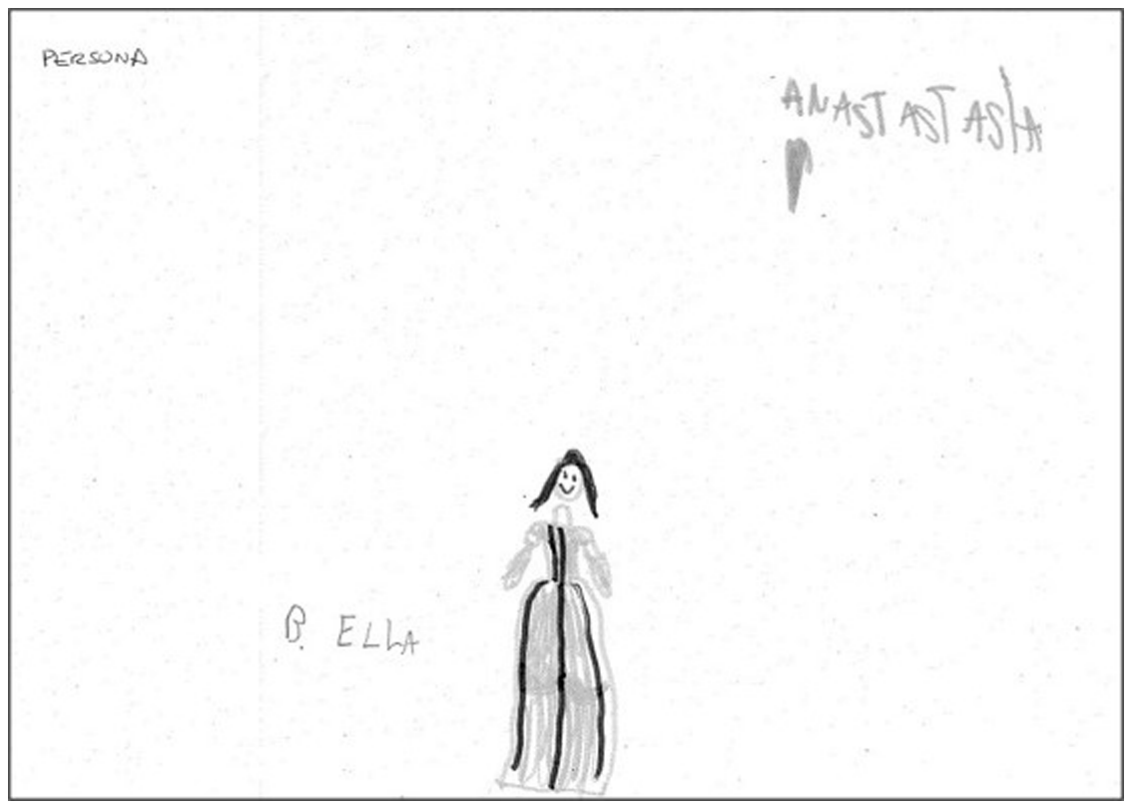
Subject 8: First drawing (female figure) of 7-year-old girl. CSA indicators: Indicator 2 (head detached from body), Indicator 4 (dot/shaded eyes), Indicator 5 (hands cut off/omitted). T2F emotional indicators: Indicator 17 (shading of body), Indicator 18 (shading of limbs), Indicator 24 (poorly integrated figure) and Indicator 26 (hands cut off).

**Figure 4:**
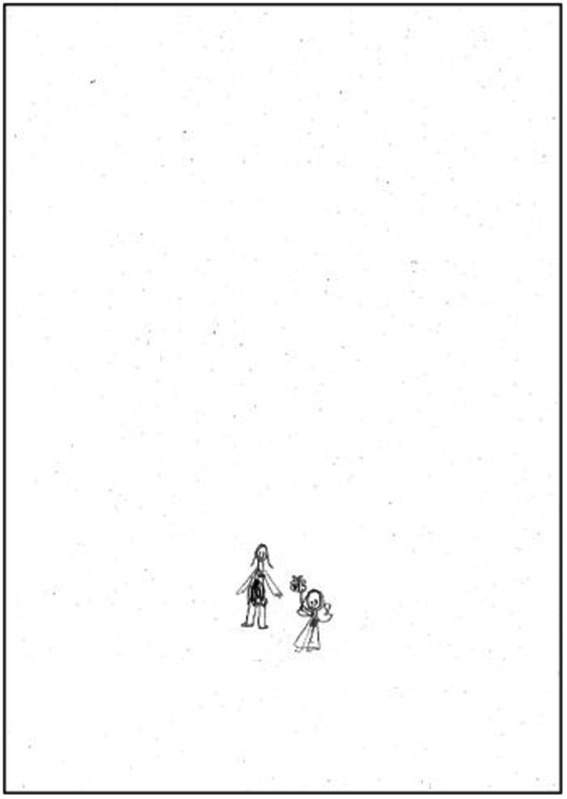
Subject 8: Second drawing (male-figure) by a 7-year-old girl. CSA indicator: Indicator 6 (genitals). T2F emotional indicators: Indicator 4 (two or more figures), Indicator 7 (transparencies), Indicator 14 (genitals or emphasised sexual features) and Indicator 17 (shading of body).

We would finally this section by illustrating these data with two human figure drawings produced by a 7-year-old girl. First, in response to the instruction to “draw a person”, she drew a female figure as a first drawing (see Figure 3) which presented three indicators of sexual abuse (CSA) and four emotional indicators (T2F). These indicators of child sexual abuse (CSA) are as follows: (2) head detached from body, (4) dot/shaded eyes, (5) hands and/or arms cut off/omitted; while the emotional indicators are (17) shading of body, (18) shading of limbs, (24) poorly integrated figure and (26) hands cut off. She was then asked to draw figure of the opposite sex, that is a male. This second drawing of the second human figure (see Figure 4) represented a male figure with genitals and added a very small female figure, with transparencies in the genital area. This male figure has one indicator of sexual abuse (CSA) and four emotional indicators (T2F). The indicator of sexual abuse (CSA) was indicator (6) genitals, while the emotional indicators were are as follows: (4) two or more figures, (7) transparencies, (14) genitals or emphasised sexual features and (17) shading of the body.

To conclude this section, we have summarised the results founds. The findings indicated the high frequency of both indicators of sexual abuse (CSA) and Emotional Indicators in Two Human Figure test (T2F) in the most human figure drawings in the sample, which confirm the existence of emotional problems. These also point out that Indicator, 1 Body omitted/distorted, is the indicator of sexual abuse CSA with the highest frequency, which was found in more than a half of the drawings in the sample. No differences based on gender were found in the study, with the exception of Indicator 1 of sexual abuse (CSA), which was more significant in boys.

## Discussion

The purpose of this study is to determine whether graphic emotional indicators are expressed in the drawings of the projective Draw-A-Person (DAP) test made by children in situations of serious risk or neglect. The results show that the two hypotheses presented in the Introduction section are confirmed.

First of all, it is important to stress that virtually the entire sample, when given the instruction to “draw a person”, drew a figure of their own sex first. That means that this test is valid and consist with the theoretical underpinnings of graphic projective techniques, which consider that the subjects project their self-concept, physical and emotional aspects, which is constructed from each individual’s subjective experiences (Hammer, 1978; Siquier de Ocampo et al., 1987; Goldner and Frid, 2021). Therefore, the human figure drawings made by the children in this study can be considered the mental picture of their self.

The first hypothesis (H1), “graphic indicators of child sexual abuse (CSA) will be found in the drawings of the sample in situations of dire risk or neglect”, is confirmed, as demonstrated by the results. Graphic indicators characteristic of child sexual abuse (CSA) was found in more than a half of the drawings by children in situations of serious risk or neglect within the sample. This is consistent with previous findings indicating the presence of specific graphic characteristics in the human figure drawings (DAP) of sexually abused children (Lev-Wiesel, 1999; Amir and Lev-Wiesel, 2007; Jacobs-Kayam et al., 2013; Goldner et al., 2021). Because the drawings in DAP tests of victims of sexual abuse have specific graphic features that differ from those drawn by persons who have not been abused (Faller, 2014; Kissos et al., 2020). As for the presence of graphic indicators characteristic of child sexual abuse (CSA) in human figure drawings, the findings of this study indicated a greater presence of three indicators of special projective significance for expressing sexual abuse: (1) body omitted or distorted, (4) eyes and (5) hands and arms, dovetailing with other studies. The first one, (1) body omitted or distorted, found in over half of the sample and more significantly in boys, that may represent anxiety about the body or certain parts of the body’s parts (Koppitz, 1968; Van Hutton, 1994; Goldner and Frid, 2021). These results are consistent with previous findings which reported that this is a frequent indicator in trauma and abused victims’ self-figure drawings. The sexual traumatization can lead to profound disturbances in the self-system, including de body image (Jacobs-Kayam et al., 2013; Dyer et al., 2015; Goldner and Frid, 2021). Furthermore, no studies have found that confirm the differences in gender and therefore these results should be dealt with cautiously and checked in subsequent studies with larger samples. The second indicator (4) eyes, could expresses a refusal to see (Colombo et al., 2004; Amir and Lev-Wiesel, 2007; Jacobs-Kayam et al., 2013). And the third indicator, (5) hands and arms, may expresses anxiety and guilt (Koppitz, 1968; Royer, 1999; Jacobs-Kayam et al., 2013; Goldner and Frid, 2021).

Moreover, Indicator (7) pre-schematic drawings, were also present showing a primitive figure that a child aged 4 or 5 would draw (Goldner et al., 2021). These drawings suggest a lack of cognitive maturity and mistreatment of children plays a crucial role in [arresting] the neurobiological and psychic maturation of individuals, and during childhood deregulates several neurobiological systems that are essential in the consolidation of complex cognitive functions and emotional regulation (Marques-Feixa and Fañanás, 2020).

The second hypothesis (H2) presented in the Introduction section, “more than a half of the participants will have a highest frequency of emotional indicators, corresponding to the upper percentiles on the emotional scale of the Two Human Figures test (T2F), confirming the existence of emotional problems”, was also confirmed. The findings point out that the vast majority of the sample has emotional problems or a high probability of having them. Bearing in mind that the children in this sample are in situations of severe risk or neglect, this is consistent with previous findings indicating the chronic experience of numerous types of mistreatments raises the risk of developing psychological and emotional problems (Warmingham et al., 2019). Moreover, the emotional indicators suggesting the presence of sexual abuse included several that strengthened the graphic indicators characteristic of child abuse (CSA) mentioned above. Specifically, indicator (7) transparencies, which graphically may represent anxiety over the body part that is transparent, which is possibly linked to some experience of mistreatment and/or abuse. Likewise, indicator (14) genitals or emphasised sexual features (Lev-Wiesel, 1999; Jacobs-Kayam et al., 2013), is also considered to be of special clinical relevance, by the T2F’s authors, which could express body-related distress associated with sexuality. The last one, indicator (21) no body, also considered to be of special clinical relevance and coincident with the indicator of sexual abuse CSA, (1) body omitted/distorted) of the first hypothesis (H1). This is one of the less frequent emotional indicators from the T2F test, as only 1.5% of the clinical subjects, with similar frequencies in both sexes, omit the body (Maganto and Garaigordobil, 2009b). These findings suggest that much of the sample studied may have experienced situations of mistreatment and/or sexual abuse.

Nevertheless, a variety of indicators is also present, such as (12) large hands or fingers, (17) shading of body, (18) shading of limbs, (22) omission of arms and (26) hands cut off. These indicators are related with the presence of anxiety over doing activities with their hands and/or arms, creating feelings of worry or guilt among the children for not behaving properly (Koppitz, 1968; Colombo et al., 2004; Pont, 2012). In addition, we found indicator (32) empty eyes, which is frequent among sexually abused children (Lev-Wiesel, 1999; Amir and Lev-Wiesel, 2007; Jacobs-Kayam et al., 2013), referring to a denial of reality, not seeing or not wanting to see. However, it should be noted that no significant differences based on gender and age were found in this study, except for the indicator of sexual abuse (body omitted/distorted), mentioned above.

Finally, we should mention the example of the two drawings (Figures 3, 4) by the 7-year-old girl (Subject 8). We can see how drawing the second human figure, in this case a male, enabled her to express the hard-to-detect abuse she had experienced. These drawings communicated the physical abuse and there are consistent with previous findings indicating that drawing encourage disclosure of disturbing content (Amir and Lev-Wiesel, 2007; Goldner et al., 2021). The fact in Catalonia (Spain), one out of every five people has suffered from some form of sexual violence in their childhood (Save the Children, 2017), and its prevalence around the world seems to concur that is occurs persistently (Pereda et al., 2009; Stoltenborgh et al., 2011; Nguyen et al., 2017). Unfortunately, it is particularly difficult to detect it in children and most of them, do not disclose their sexual abuse during childhood (Read et al., 2017; Faller, 2020). Difficulties in detection are keeping child mistreatment and abuse hidden from the public eye (Tello, 2020). To have tools like the DAP are needed to improve in childhood mistreatment and abuse detection.

## Conclusions and limitations

The results of this study suggest that the human figure drawings (DAP), and especially the two human figures (T2F) projective test, facilitate the externalisation of traumatic situations of mistreatment and/or abuse experienced by children. Moreover, these findings have important implications for professionals, as the use of this projective technique can help to alert and to identify aspects of risky situations, and in turn, it can help in the design of global intervention strategies in children and their families in situations of mistreatment and/or abuse.

Nevertheless, several limitations of this study should be taken into consideration. First, the participants are only from one urban area, Barcelona, and one country, Spain. Future studies replicating the findings with an expanded sample, including subjects from different countries, are needed. This would enable the results from this study to be checked and validated. Second, our results are based solely on drawings. Future studies, should include additional measures such as narratives, which would be values complement to drawings, allowing other relevant variables such as attachment in child abuse to be evaluated (Fresno et al., 2018; Muzi et al., 2021). Finally, our findings concentrated on drawings of the projective Draw-A-Person test (DAP). Future research could use other drawings tools at the same time, such as Family Drawings (FD), to assess attachment representations as a cross-cultural method (Pace et al., 2022a). These could provide to further explore the children’s experiences of mistreatment and/or sexual abuse in other cultures.

## Data availability statement

The original contributions presented in the study are included in the article/supplementary material, further inquiries can be directed to the corresponding author.

## Ethics statement

The project was approved by the Ethics and Research Committee at the Universitat Ramon Llull (URL) in Barcelona (Spain). Written informed consent was obtained from the participants legal guardian/next of kin.

## Author contributions

EB is the principal researcher, conceptualized, structured data and writing most part of the manuscript. M^a^C and M^a^P contributed to documenting and writing data of the Child Protection section in Catalonia and the SEAIA teams of Barcelona City Council. PB organized the sample data, analyzed the statistical data and performed the Tables. All authors contributed to the article and approved the submitted version.

## Acknowledgments

We thank the children who participated in the study. We also thank our colleague of FPCEE Blanquerna (URL), Vidal Sanchis, for his collaboration in organizing the drawings and the sample’s data.

## Conflict of interest

The authors declare that the research was conducted in the absence of any commercial or financial relationships that could be construed as a potential conflict of interest.

## Publisher’s note

All claims expressed in this article are solely those of the authors and do not necessarily represent those of their affiliated organizations, or those of the publisher, the editors and the reviewers. Any product that may be evaluated in this article, or claim that may be made by its manufacturer, is not guaranteed or endorsed by the publisher.
